# Supplementary material for: Optimized Production of a Redox Metabolite (pyocyanin) by Pseudomonas aeruginosa NEJ01R Using a Maize By-Product
Source: Microorganisms. 2020 Oct 10;8(10):1559. doi: 10.3390/microorganisms8101559 (PMC7599481; doi:10.3390/microorganisms8101559)
Supplement: Supplementary file 1 [file microorganisms-08-01559-s001.zip › microorganisms-915050-sup/Bacame et al SM.doc]

**Table S1.** Experimental design matrix for biomass and PYO production in defined culture medium.

| **Experimental factors** | **Levels** | | | | |
| --- | --- | --- | --- | --- | --- |
|  | -1.4711 | -1 | 0 | 1 | 1.4711 |
| Temperature, °C (X_1_) | 21.5 | 25 | 32.5 | 40 | 43.5 |
| pH (X_2_) | 5 | 6 | 8 | 10 | 11 |
| Velocity of agitation, rpm (X_3_) | 76 | 100 | 150 | 200 | 224 |

**Figure S1. a)** Chromatograms and absorption spectra of PYO standard and **b)** PYO extracted from *P. aeruginosa* NEJ01R culture supernatant.

**Figure S2. a)** Mass spectra of PYO standard and **b)** PYO extracted from *P. aeruginosa* NEJ01R culture supernatant**.**
